# Delayed Presentation of a Post-traumatic Suprascapular Artery Pseudoaneurysm

**DOI:** 10.7759/cureus.55618

**Published:** 2024-03-06

**Authors:** Robert Myers, Bakhtawar Mushtaq, Robert Jubelirer, Orlando Kirton

**Affiliations:** 1 General Surgery, Abington Jefferson Hospital, Abington, USA

**Keywords:** angiogram, pseudoaneurysm, computed tomography, suprascapular, trauma

## Abstract

Thyrocervical trunk pseudoaneurysms are a rare entity among pseudoaneurysms, mostly caused by trauma. We present the case of a 74-year-old male who suffered a traumatic pseudoaneurysm of the supra-scapular artery after a rib and scapular fracture. The patient was treated with various interventions along the treatment algorithm, including ultrasound-guided thrombin injection, coil embolization, and surgical excision. In our patient, the pseudoaneurysm was successfully treated with coil embolization, but a persistent thrombosed pseudoaneurysm caused the patient discomfort, prompting the eventual surgical removal. This case is unique as it enlightens the step-wise approach to managing thyrocervical trunk pseudoaneurysm.

## Introduction

A pseudoaneurysm, also known as a false aneurysm, is defined as arterial extravasation after disruption of the arterial wall. The pseudoaneurysm is surrounded mainly by tunica adventitia instead of all the three vessel layers (intima, media, adventitia), as is seen in a true aneurysm. Pseudoaneurysm arising from the thyrocervical trunk is a rare occurrence [1-3].

The suprascapular artery is the first branch of the thyrocervical trunk and courses superior and posterior to the clavicle. It supplies supra- and infraspinatus muscles [4]. Given the precarious path it takes to reach its intended organs, the suprascapular artery is at risk of injury from the surrounding elements. Summers et al. described a case of suprascapular artery aneurysm from continuous wear and tear and vascular injury from glenohumeral joint osteoarthritis [4]. 

Most commonly, cases of pseudoaneurysms are attributable to direct trauma, central venous cannulation, repetitive overhead arm motions in select athletes, or thoracic outlet syndrome [2,3,5]. Bellenot et al. mentioned congenital causes, such as an arteriovenous malformation leading to a Cirsoid aneurysm (arteriovenous malformation of scalp and extremities) and connective tissue disorders such as Ehlers-Danlos syndrome, have also been identified [6].

## Case presentation

A 74-year-old male with a medical history of hypertension and hyperlipidemia presented to our trauma center following a mechanical fall down a flight of stairs, which was followed by immediate pain in his back and right shoulder. He was hemodynamically normal on presentation, and his trauma work-up (CT of the head, cervical spine, chest abdomen, and pelvis) was remarkable for the right scapular fracture, six right-sided rib fractures, and two left-sided rib fractures. There were no clinical signs of vascular injury at the time of presentation. He was observed in the intensive care unit for one day, then transferred to the floor, and subsequently discharged home the next day with an uncomplicated hospital course. 

**Figure 1:**
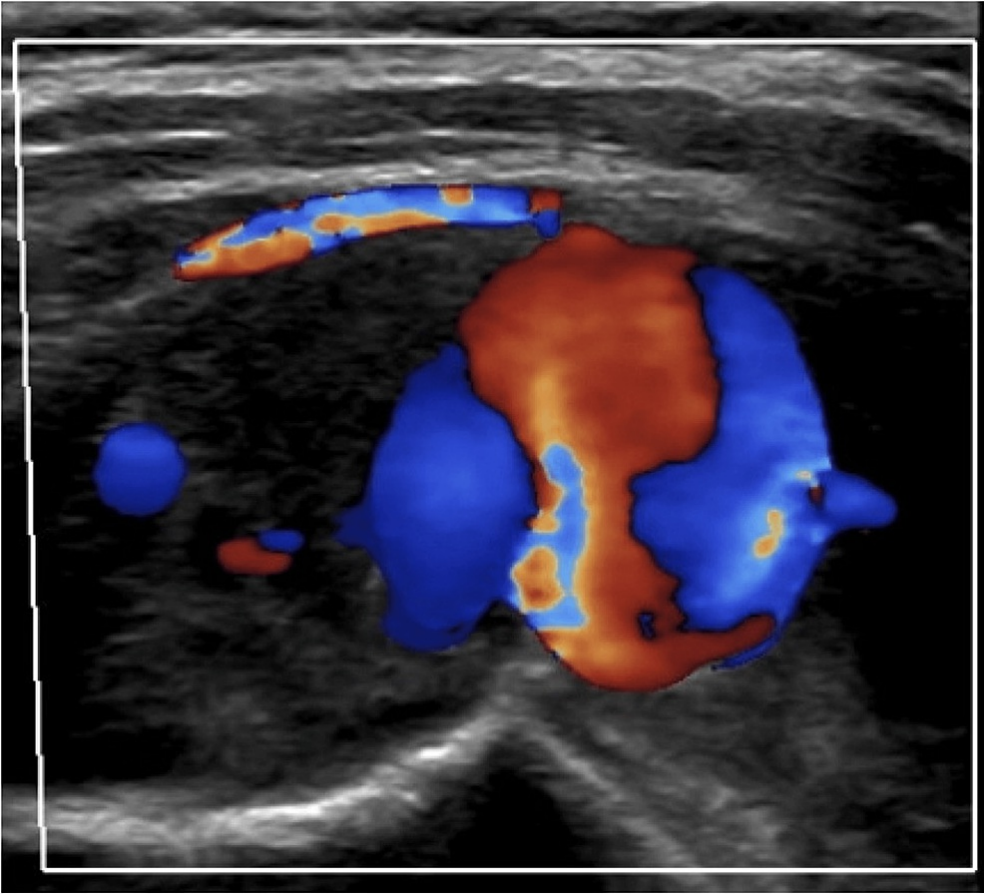
Ultrasonographic demonstration of heterogeneous hypoechoic mass with central vascular flow

He was seen by his primary care physician about 10 months after his initial presentation due to discomfort from a palpable, non-tender, non-erythematous mass on his right upper back. He underwent ultrasonography with Doppler of the mass, and the findings were significant for a 4.5x4.5x1.2 cm heterogeneous hypoechoic mass with the central vascular flow that demonstrated arterial waveforms concerning partially thrombosed pseudoaneurysm (Figure 1).

He subsequently underwent imaging with a CT angiogram of the chest, which confirmed the pseudoaneurysm.

**Figure 2:**
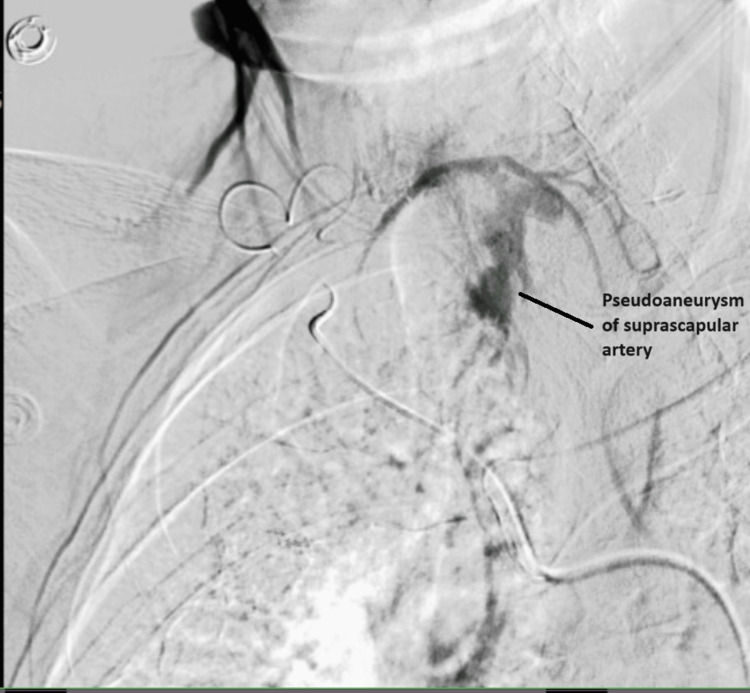
Angiography of subclavian artery with the demonstration of pseudoaneurysm of suprascapular artery

He was referred to interventional radiology, who performed a successful ultrasound-guided thrombin injection (~3000 units) of the pseudoaneurysm with rapid thrombosis. At his three-month post-procedure follow-up, his ultrasound re-demonstrated the partially thrombosed pseudoaneurysm sac with inner core flow measuring 1.4x1.9x2.4 cm. A subsequent right upper extremity arteriogram with a focus on the subclavian artery demonstrated the vessel to be patent, leading into the thyrocervical trunk and supplying the pseudoaneurysm via the suprascapular artery (Figure 2).

**Figure 3:**
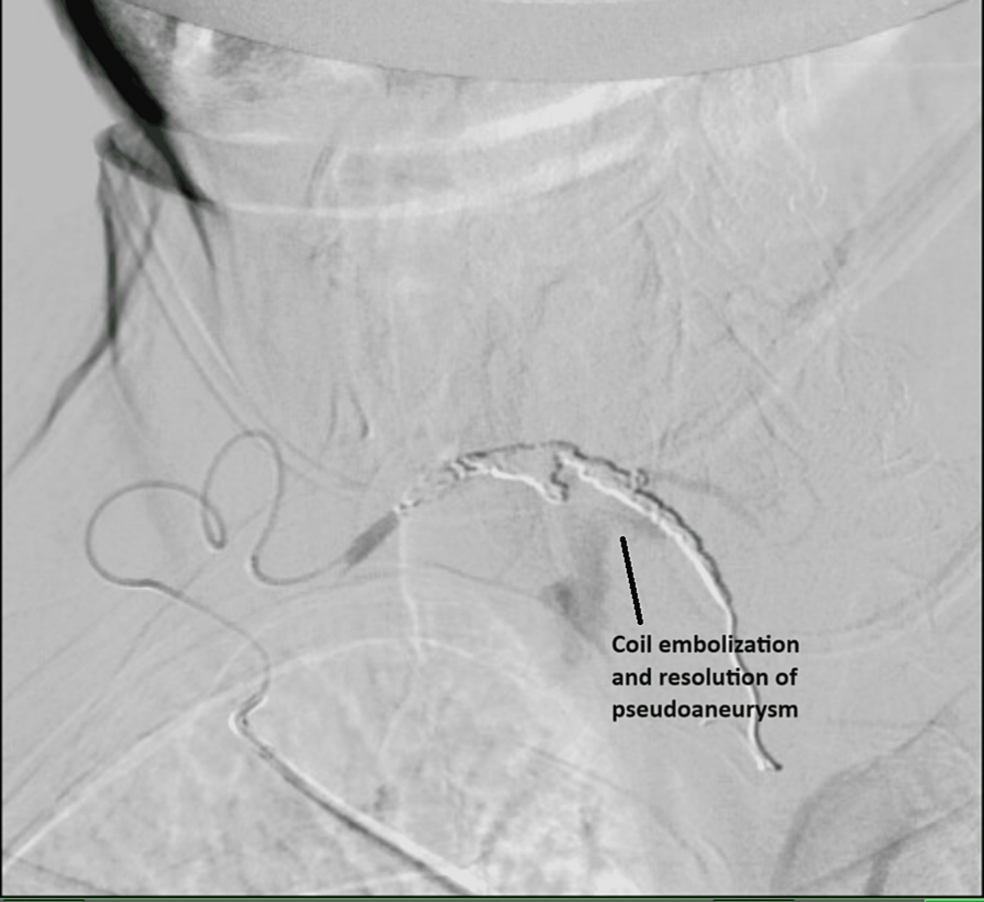
Imaging demonstrating successful coil embolization

The pseudoaneurysm was successfully coil embolized under fluoroscopic guidance (Figure 3).

He subsequently followed up three months later with an ultrasound, which demonstrated a completely thrombosed pseudoaneurysm. Due to persistent pain and discomfort while supine, he was referred to the vascular surgery office and underwent surgical excision of the thrombosed pseudoaneurysm in the outpatient setting. He tolerated the procedure well and has been asymptomatic since.

## Discussion

Pseudoaneurysms of the upper extremity are less common when compared to those in the lower extremity [1]. Furthermore, pseudoaneurysms of the thyrocervical trunk and its branches are very rarely encountered. When present, the inferior thyroid artery is most commonly affected, with only 15 reported cases since 1959. Pseudoaneurysm of the suprascapular artery is even rarer and is mostly attributed to repetitive overhead movements, which cause arterial thoracic outlet obstruction secondary to a cervical rib or trauma [3,5,7]. Penetrating trauma is most commonly responsible for arterial injury secondary to bone fragments from scapular, clavicle, or rib fractures, as in our patient. The most common presentation is a palpable pulsatile mass, which can cause discomfort while lying down, as with our patient, depending on the size and location of the lesion. If there is concern of infection, it presents as a tender, warm, and erythematous mass.

Since trauma is the most common cause, it's important to evaluate patients with high-energy trauma with a complete workup that includes computed tomography (CT) of the head, neck, chest-abdomen, and pelvis based on the mechanism. Patients with clavicle fractures and first rib fractures are more at risk of subclavian artery injury; patients should be monitored for late vascular injury even if no injury is present at the initial workup. Several imaging modalities are used to identify pseudoaneurysms. Noninvasive modalities include Duplex sonography, CT angiography, and magnetic resonance (MR) angiography. Duplex ultrasound has a 94% sensitivity and 97% specificity for the identification of superficial pseudoaneurysms [8]. The typical appearance of a pseudoaneurysm on ultrasound is a "to-and-fro" pattern, documenting the turbulent flow within the lesion [8,9]. Thin slice CT angiography has a sensitivity and specificity of 95.1% and 98.7%, respectively, in detecting pseudoaneurysms [10].

Among invasive diagnostic modalities, catheter-directed angiography remains the gold standard for the identification of a pseudoaneurysm. It has the benefit of providing a clear depiction for identifying collateral vessels and options for therapeutic endovascular intervention [10]. 

The procedure of choice depends on the resources available and the expertise of the surgeon or endovascular specialist. Treatment options include surgical resection, ultrasound-guided thrombin injection, or catheter director coil embolization [11]. Minimally invasive techniques are usually attempted first, giving excellent results and fast recovery. Surgical resection is considered the gold standard, given the anatomic location of the aneurysm. In our case, we present the treatment plan of attempting interventional procedures before attempting open surgical intervention. As we did with our patient, one can try an ultrasound-guided thrombin injection with a follow-up ultrasound in three months to confirm the resolution of the pseudoaneurysm. If the pseudoaneurysm is not completely resolved, the next least invasive step is coil embolization by interventional radiology. If the pseudoaneurysm is not able to be reached with wires due to anatomical constraints or after embolization, the patient remains symptomatic, then the most invasive approach of surgical excision is required. As seen with our patient, the procedure is generally well tolerated, with a timely return to baseline functioning.

## Conclusions

Trauma has been attributed as the most common cause of pseudoaneurysm of the upper extremity. Diagnostic modalities range from non-invasive techniques, including Duplex sonography, CT angiography, and MR angiography to invasive conventional angiography. Conventional angiography is the gold standard modality given the dual benefit of diagnosing and treatment of the pseudoaneurysm. The stepwise approach to treatment is similar to a pseudoaneurysm of the upper and lower extremity. It includes minimally invasive interventional procedures (ultrasound-guided thrombin injection, coil embolization) that can be trialed before pursuing definitive open surgical therapy.
